# Analysis of the interaction between Zinc finger protein 179 (Znf179) and promyelocytic leukemia zinc finger (Plzf)

**DOI:** 10.1186/1423-0127-20-98

**Published:** 2013-12-20

**Authors:** Ding-Yen Lin, Chi-Chen Huang, Ya-Ting Hsieh, Hsin-Chuan Lin, Ping-Chieh Pao, Jen-Hui Tsou, Chien-Ying Lai, Liang-Yi Hung, Ju-Ming Wang, Wen-Chang Chang, Yi-Chao Lee

**Affiliations:** 1 Institute of Bioinformatics and Biosignal Transduction, College of Bioscience and Biotechnology, National Cheng Kung University, Tainan 70101, Taiwan; 2 Ph.D. Program for Neural Regenerative Medicine, College of Medical Science and Technology, Taipei Medical University, Taipei 11031, Taiwan; 3 Center for Neurotrauma and Neuroregeneration, Taipei Medical University, Taipei 11031, Taiwan; 4 Graduate Institute of Medical Sciences, College of Medicine, Taipei Medical University, Taipei 11031, Taiwan

**Keywords:** Zinc finger protein 179, Ring finger protein 112, Promyelocytic leukemia zinc finger, RING finger

## Abstract

**Background:**

Zinc finger protein 179 (Znf179), also known as ring finger protein 112 (Rnf112), is a member of the RING finger protein family and plays an important role in neuronal differentiation. To investigate novel mechanisms of Znf179 regulation and function, we performed a yeast two-hybrid screen to identify Znf179-interacting proteins.

**Results:**

Using a yeast two-hybrid screen, we have identified promyelocytic leukemia zinc finger (Plzf) as a specific interacting protein of Znf179. Further analysis showed that the region containing the first two zinc fingers of Plzf is critical for its interaction with Znf179. Although the transcriptional regulatory activity of Plzf was not affected by Znf179 in the Gal4-dependent transcription assay system, the cellular localization of Znf179 was changed from cytoplasm to nucleus when Plzf was co-expressed. We also found that Znf179 interacted with Plzf and regulated Plzf protein expression.

**Conclusions:**

Our results showed that Znf179 interacted with Plzf, resulting in its translocation from cytoplasm to the nucleus and increase of Plzf protein abundance. Although the precise nature and role of the Znf179-Plzf interaction remain to be elucidated, both of these two genes are involved in the regulation of neurogenesis. Our finding provides further research direction for studying the molecular functions of Znf179.

## Background

Znf179, also known as Rnf112, is a RING finger protein with a characteristic C3HC4-type Zinc-finger motif located in the N-terminus. The expression of Znf179 is abundant in brain and is regulated during brain development [1,2], suggesting a potential role in nervous system development. Our previous study has first revealed the cellular function of Znf179 in neuronal differentiation. We demonstrated that induction of the *Znf179* regulated p35 expression and accumulation of p27 protein, which led to cell cycle arrest in G0/G1 phase, and was critical for neuronal differentiation [3]. The human *ZNF179* gene is located on chromosome 17p11.2 and is present in the Smith-Magenis syndrome (SMS) common deletion region [4]. Therefore, *ZNF179* is considered to be one of the candidate genes for SMS, which is a complex neuropediatric-neurobehavioral syndrome [1,5]. In addition, previous studies using a microarray analysis have demonstrated that Znf179 is significantly down-regulated in neurodegenerative diseases such as Huntington’s disease (HD) and amyotrophic lateral sclerosis (ALS), implying that Znf179 may associate with neurodegenerative diseases [6,7]. However, to date, the function and the molecular mechanisms of Znf179 in neural development and disease progression remain mostly unknown.

The promyelocytic leukemia zinc finger (*Plzf*) is a *kruppel*-like C2H2 zinc finger gene which is previously identified in a rare case of acute promyelocytic leukemia (APL) with a variant chromosomal translocation t(11:17) (q23;q21) and resistance to therapy with *all-trans*-retinoic acid (ATRA) [8]. Plzf is a transcriptional repressor that binds to the promoter of various genes, such as cyclin A2 and c-*myc* through its *kruppel*-like zinc fingers [9-11]. Plzf also contains an N-terminal BTB/POZ domain, which is a conserved structural motif found in a number of pox and zinc-finger proteins, and has been shown to mediate homo/heterodimerization, nuclear localization as well as to direct binding of corepressors [12,13]. It has been found that the Plzf can repress transcription through recruitment of nuclear receptor corepressors (N-CoR or SMRT)/histone deacetylase (HDAC) complexes via its POZ domain [14]. In addition, Plzf is also able to activate gene expression [15,16]. The physiological function of Plzf is the maintenance of stem cells of various lineages, such as hematopoietic stem cells and spermatogonial stem cells, and is implicated in embryonic development and hematopoiesis [17,18]. Disruption of *Plzf* in mice leads to defect in spermatogenesis and patterning of the limb and axial skeleton [19-21]. Although the functional role of Plzf in brain development is less studied, Plzf is expressed in spatially restricted and temporally dynamic patterns in the central nervous system. During mouse embryogenesis, expression of Plzf is found in the anterior neuroepithelium at early stage (E7.5) and extends to entire neuroectoderm until stage E10 [22,23]. Recently, Plzf has been found to inhibit neurogenesis in Zebrafish [24]. Taken together, Plzf has been implicated in hematopoietic, spermatogonial stem cells maintenance and in inhibition of neurogenesis.

Here we demonstrated a physical and functional interaction between Znf179 and the Plzf. Plzf altered the subcellular localization of Znf179. Additionally, Znf179 regulated the protein levels of Plzf. Our findings provide possible function of Znf179 and highlight a potential research direction for studying the molecular functions of Znf179.

## Methods

### Plasmid construction

A PCR fragment encoding the N-terminal (amino acids 1–417) of Znf179 was subcloned into vector pBTM116 in-frame with LexA to generated the LexA-Znf179 (1–417) bait. pGal-AD-Plzf deletion mutants were engineered by subcloning PCR-amplified Plzf fragments into the yeast vector pACT2, which expresses the Gal4 activation domain (BD Biosciences Clontech, Palo Alto, CA, USA). To generate Znf179 and Plzf expression vectors for mammalian cells, the full-length or partial cDNA fragments were amplified by PCR using IMAGE clone 4506141 (GeneBank entry BC037118) and 4944546 (GeneBank entry BC026902) as templates, respectively. Sequences of the primers used were listed in Additional file 1: Table S1. EGFP-Znf179, EGFP-Znf179 (1–153) and EGFP-Znf179 (154–654) were generated by inserting Znf179 cDNA fragments into pEGFP vector (Clontech). Flag-Plzf, Flag-Plzf (1–398), Flag-Plzf (180–673), Flag-Plzf (398–673), Flag-Plzf (455–673) and Flag-Plzf (515–673) were generated by inserting Plzf cDNA fragments into pCMV-Tag2 vector (Stratagene, La Jolla, CA, USA). The full-length cDNA fragments of Znf179 and Plzf were also inserted in-frame into the pM vector (Clontech), a vector for the expression of GAL4 DBD (DNA binding domain) fusion proteins from a constitutive SV40 (simian virus 40) early promoter. The constructs of HA-Plzf and Arora kinase C promoter were described elsewhere [25]. pFR-Luc reporter plasmid (Stratagene, La Jolla, CA, USA) contains a synthetic promoter with five tandem repeats of the yeast GAL4 binding elements that control expression of the firefly luciferase gene. pRL-TK, a plasmid contains the Renilla luciferase as transfection control, was purchased from Promega (Madison, WI, USA).

### Yeast two-hybrid screen and β-galactosidase activity assay

The LexA-Znf179 (1–417) construct was used to screen against with mouse brain cDNA library (Clontech). Yeast two-hybrid screen was performed as described previously [26]. L40 yeast strain was first transformed with LexA-Znf179 (1–417), followed by 100 μg of the brain cDNA library transformation. The library of transformants was selected on medium lacking histidine, leucine, and tryptophan. His^+^ colonies were further tested for β-galactosidase activity using a colony lift filter assay. The plasmids from both of His^+^ and X-gal^+^ colonies were isolated by the curing process of MC1066 bacterial strain and retransformed with LexA-Znf179 (1–417) or LexA-lamin to test the binding specificity. The library plasmids conferred that the Znf179-specific interactions were then subjected to DNA sequence analysis. Quantitative X-gal assays were performed with yeasts containing pairs of bait and prey plasmids as indicated. The X-gal activities were determined from three separate liquid yeast cultures as described previously [27].

### Cell culture

COS-1 and HeLa cells were cultured in Dulbecco’s modified Eagle’s medium (DMEM; Invitrogen, Carlsbad, CA, USA) supplemented with 10% fetal bovine serum (FBS; Invitrogen). SW480 cells were cultured in Leibovitz’s L-15 medium (Invitrogen) supplemented with 10% FBS. P19 cells were maintained in alpha minimum essential medium (α-MEM; Invitrogen) supplemented with 7.5% bovine serum (BS; Invitrogen) and 2.5% FBS. All cells were maintained at 37°C under a 5% CO2 atmosphere. To induce P19 cells differentiation, cells were allowed to aggregate in bacterial-grade Petri dishes at a seeding density of 1 × 10^5^ cells/ml in the presence of 1 μM all-*trans*-RA (Sigma-Aldrich). After 4 days of aggregation, cells were dissociated into single cells by trypsin-EDTA, and were plated in a poly-L-lysine-coated tissue culture dish at a density of 1 × 10^5^ cells/cm^2^ in Neurobasal^TM^-A medium (Invitrogen) with a 1× B27 supplement. Cells were allowed to attach for 24 h, and then were exposed to 10 μM Ara-C 24 h to inhibit proliferation of non-neuronal cells.

### Antibodies

The following antibodies were used for the Western blot, immunoprecipitation, and immunofluorescence analyses: Plzf (Santa Cruz, Santa Cruz, CA, USA), HA (Covance, Princeton, NJ, USA), Flag (Sigma-Aldrich, St. Louis, MO, USA) and EGFP (Clontech). The polyclonal Znf179 antibodies were generated against a synthetic peptide (CEKEEDERVQGGDREPLLQEE) corresponding to C terminal amino acids 634 ~ 654 of mouse Znf179 [3].

### Immunoprecipitation

For testing the association of Znf179 and Plzf in mammalian cells, EGFP-Znf179 were co-transfected with Flag-Plzf construct into HeLa cells. Forty-eight hours after transfection, cells were solubilized in 1 ml of lysis buffer, containing 50 mM Tris–HCl (pH 7.8), 150 mM NaCl, 15 mM EDTA, 0.5% Triton X-100, 0.5% Nonidet P-40, and 0.1% sodium deoxycholate and Complete^TM^ Protease Inhibitor Cocktail (Roche Applied Science, Indianapolis, IN, USA). Whole cell lysates were mixed with antiserum against Flag, and the immunocomplexes were mixed with protein A-Sepharose beads (GE Healthcare Life Sciences Buckinghamshire, UK). After 2 h incubation, the immunocomplexes were then gently washed three times with the same buffer as described above followed by Western blot analysis with the anti-Flag and anti-EGFP antibodies.

### Immunofluorescence

Cells were fixed for 15 min with 4% formaldehyde in phosphate-buffered saline (PBS) and then permeabilized with cold acetone. Antibodies were then incubated with fixed cells for 4 h at room temperature. Cells were washed three times with PBS followed by incubation with a secondary antibody for 1 h at room temperature. Nuclei were revealed by ProLong® Gold antifade reagent with DAPI (Invitrogen). Coverslips were inverted, mounted on slides, and sealed with nail polish. Pictures were taken using fluorescence microscopy.

### Transfection and reporter activity assays

Transfection-grade DNA is prepared using PurelinkTM HiPure kits (Invitrogen). All of the transfections were performed by using Lipofectamine 2000^TM^ (Invitrogen). After 24 h, cell lysates were prepared and reporter activities were measured by the Dual Luciferase Reporter kit (Promega). The assay was performed according to manufacturer’s recommendations, and luciferase activity was measured with Triathler Multilabel Tester 1.9 (Hidex, Oy, Turku, Finland). The transfection efficiency was corrected by normalizing the data to the corresponding *Renilla* luciferase activity for each construct.

### Reverse transcription (RT) and quantitative (q) real-time PCR assays

Total RNA was extracted using the Trizol reagent (Invitrogen) following the manufacturer’s recommendations. RNA was then treated with DNase I (Ambion, Austin, Texas, USA) to remove DNA contamination. RT was performed with 1.5 μg of total RNA using M-MLV reverse transcriptase (Invitrogen). A real-time qPCR was performed using the SYBR advantage qPCR premix (Invitrogen). The PCRs were then performed using the following conditions for 40 cycles: 95 1C for 15 s, 60 1C for 15 s, and 72 1C for 20 s. The sequences of primers used for RT-PCR were as follows: Plzf forward, 5′-CCACCTTCGCTCACATACAG-3′, reverse, 5′-TCTTGCCACAGCCATTACA-3′; and β-actin forward, 5′-ACTGGGACGACATGGAGAAG-3′, reverse, 5′-GGTACGACCAGAGGCATACAG-3′. Real-time fluorescence monitoring and a melting-curve analysis were performed with LightCycler according to the manufacturer’s recommendations (Roche Molecular Diagnostics, Lewes, East Sussex, UK). Negative controls containing no cDNA template were included in each experiment. A melting curve was created at the end of the PCR cycle to confirm that only a single product was amplified. Data were analyzed by LightCycler software version 3.5 (Roche Molecular Diagnostics) to determine the threshold cycle (Cp) above the background for each reaction. The relative transcript amount of the target gene, calculated using standard curves of serial cDNA dilutions, was normalized to that of β-actin of the same cDNA.

## Results

### Identification of Plzf as a Znf179-interacting protein

**Figure 1:**
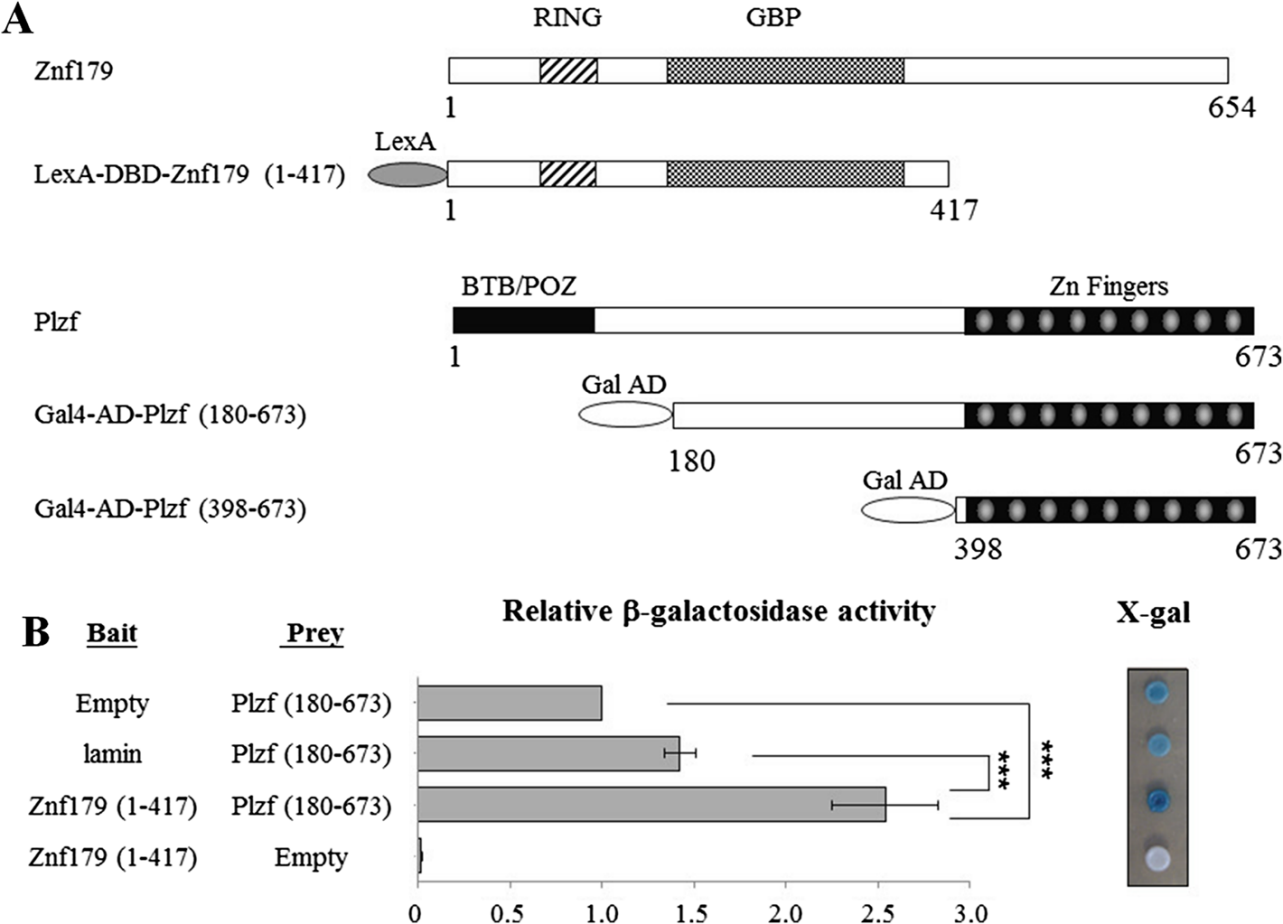
**Interaction of Plzf and Znf179 in yeast two-hybrid assay. (A)** Schematic representation of Plzf clone that interacts with LexA-Znf179 (1–417) bait. N-terminal fragment of Znf179 was tagged at the N terminus with LexA. The Plzf fragment represents the clone isolated from the yeast two-hybrid screen of a mouse brain library linked to the Gal4 transactivation domain (Gal-AD). The first and last amino acids of the fragments are numbered with respect to their positions in full-length protein. **(B)** Yeast strain L40 was co-transformed with a bait (Znf179 N terminal fragment or the control protein lamin fused to the LexA protein) and a prey protein (Plzf fragment 180–673 fused to the Gal-AD or Gal-AD alone). Interaction was monitored by β-galactosidase activity assay using o-nitrophenylgalactoside or X-gal as substrates. Values represent the mean ± S.E.M. of at least three independent experiments. Statistical analysis was performed using one-way ANOVA with appropriate post hoc tests: ***P < 0.001.

To identify Znf179-interacting proteins, a yeast two-hybrid screen was undertaken by using the mouse Znf179 N-terminal fragment (amino acids 1–417) as a bait in a LexA-based two-hybrid system together with a mouse brain cDNA library (Figure 1A). From the screening, 17 positive clones were obtained and all were identified to encode the same protein. Sequence analyses revealed that the inserts from each individual clone corresponded to the promyelocytic leukemia zinc-finger (Plzf) protein with two different fragments (amino acids 180–673 and 398–673). To verify the interaction between Znf179 and Plzf in yeast, we transformed Gal4-Plzf (180–673) with LexA-Znf179 (1–417) or control vector, and found that Plzf had an autonomous activating activity (Figure 1B), which was previously reported [27]. We therefore measured the β-galactosidase activity quantitatively by liquid β-galactosidase assay. The results showed that the β-galactosidase activity in yeast strain containing LexA-Znf179 (1–417) and Gal4-Plzf (180–673) was significantly higher than that containing LexA-lamin (as negative control) and Gal4-Plzf (180–673) or Gal4-Plzf (180–673) alone (Figure 1B).

**Figure 2:**
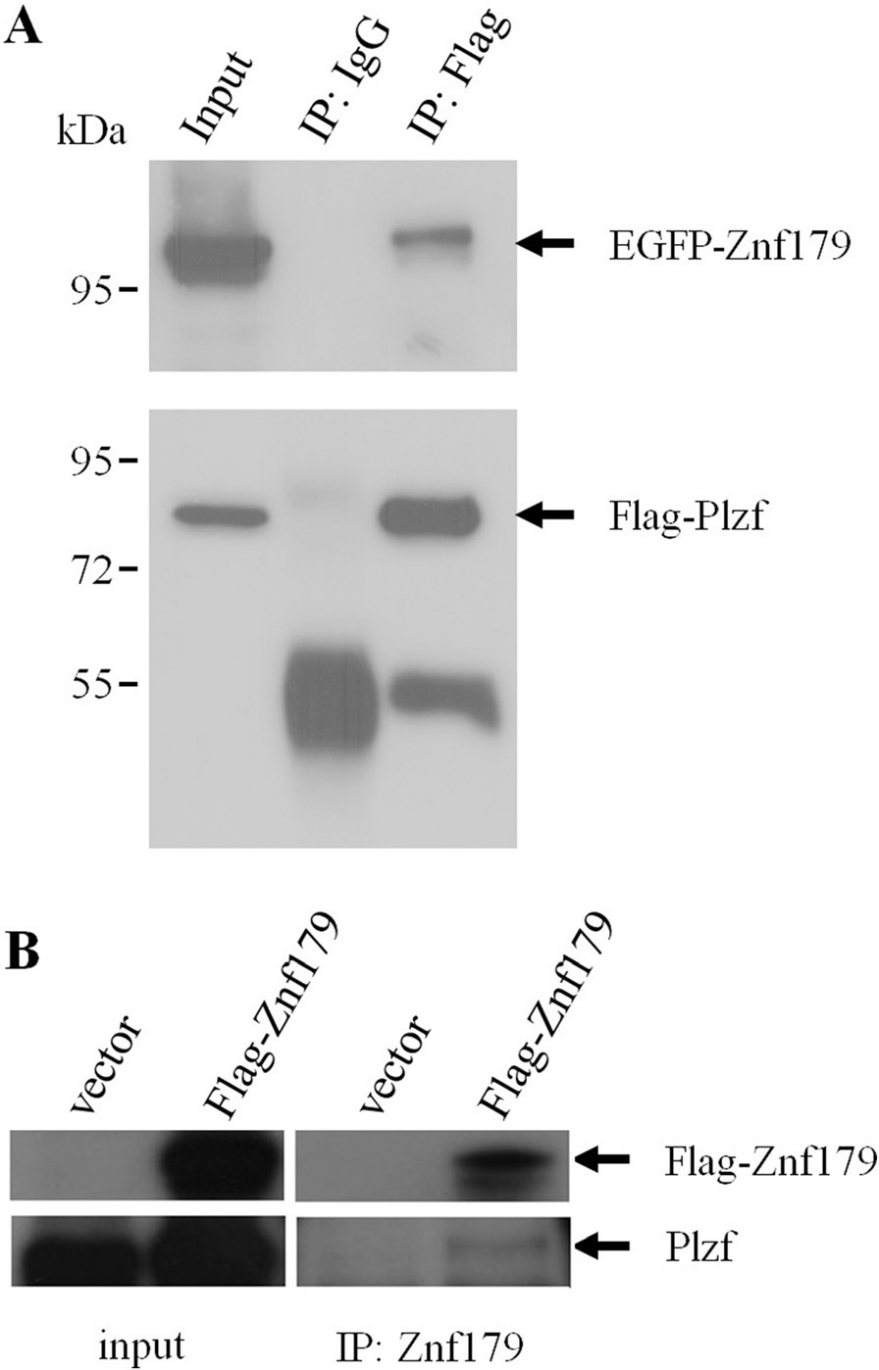
**Plzf interacts with Znf179 *in vivo*. (A)** COS-1 cells were co-transfected with Flag-Plzf and EGFP-Znf179 vectors. Immunoprecipitation (IP) was performed with anti-Flag antibody or mouse IgG. The protein complex was then washed and analyzed by Western blotting as indicated. **(B)** P19 cells were transfected with Flag-Znf179 or vector only, then aggregation was induced in the presence of 1 μM all-*trans*-RA for two days. IP was performed with anti-Znf179 antibody. The protein complex was then washed and analyzed by Western blotting as indicated.

To further confirm the protein interaction between Znf179 and Plzf, the full-length Znf179 and Plzf cDNAs were amplified by PCR using IMAGE clone 4506141 (GeneBank entry BC037118) and 4944546 (GeneBank entry BC026902) as templates, respectively. The derived Znf179 and Plzf cDNAs were subcloned in-frame into the pEGFP-C and pCMV-Tag vectors, respectively. To establish whether Plzf interacted with Znf179 in mammalian cells, cell lysate from COS-1 cells overexpressing Flag-Plzf and EGFP-Znf179 were immunoprecipitated with anti-Flag antibody followed by Western blot analysis with anti-Znf179 antibody. As shown in Figure 2A, Znf179 was detected in the immunoprecipitated complexes of Plzf. The immunoprecipitation results together with the yeast two-hybrid studies provided evidence of Znf179 indeed interacted with Plzf. To further examine whether Znf179 interacted with endogenous Plzf protein, Flag-Znf179 was transfected into P19 cells and the transfected P19 cells were aggregated in the presence of 1 μM RA for 2 days. Our unpublished data showed that Plzf can be induced 2 days after aggregates induction in the presence of 1 μM RA (data not shown). The cell lysate was immunoprecipitated with anti-Znf179 antibody followed by Western blot analysis. As shown in Figure 2B, endogenous Plzf was detected in the immunoprecipitated complexes with Flag-Znf179. Our result reveals that Znf179 can interact with the endogenous Plzf protein.

### Mapping the sites of interaction between Znf179 and Plzf

**Figure 3:**
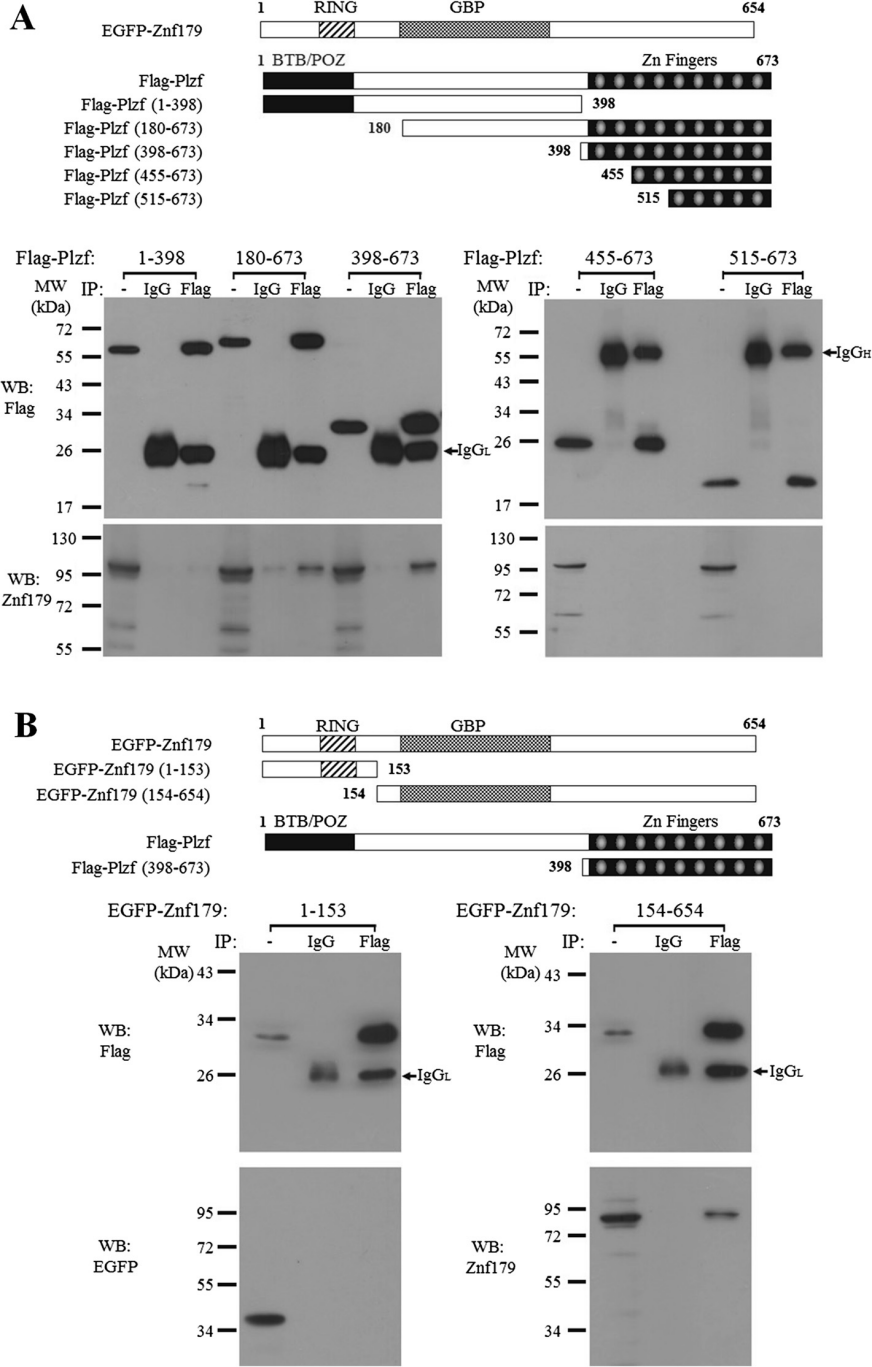
**Mapping the regions of interaction between Znf179 and Plzf. (A)** The top panel shows the schematic representation of Znf179 and various deletion constructs of Plzf used in the immunoprecipitation experiments. The first and last amino acids of the fragments are numbered with respect to their positions in full-length protein. Bottom panel: COS-1 cells were co-transfected with EGFP-Znf179 and different fragment of Flag-Plzf. IP was performed with anti-Flag antibody or mouse IgG. The protein complex was then washed and analyzed by Western blotting (WB) with the anti-Flag and anti-EGFP antibodies. **(B)** The top panel shows the schematic representation of Plzf and various deletion constructs of Znf179 used in the immunoprecipitation experiments. The first and last amino acids of the fragments are numbered with respect to their positions in full-length protein. Bottom panel: COS-1 cells were co-transfected with Flag-Plzf and different fragment of EGFP-Znf179. IP was performed with anti-Flag antibody or mouse IgG. The protein complex was then washed and analyzed by Western blotting (WB) with the anti-Flag and anti-EGFP antibodies. IgG_L_, immunoglobulin light chain; IgG_H_, immunoglobulin heavy chain.

To determine the region(s) in Plzf that was required for its interaction with Znf179, various deletion constructs of Plzf were generated and cotransfected with EGFP-Znf179 into COS-1 cells. Cell lysates were immunoprecipitated with anti-Flag antibody, followed by Western blot analysis with anti-Znf179 antibody. As shown in Figure 3A, two fragments of Plzf (amino acids 180–673 and 398–673) interacted with Znf179, which was consistent with the findings in yeast two-hybrid assay. In contrast, the N-terminal fragment (amino acids 1–398) and the last seven zinc-fingers (amino acids 455–673) of Plzf did not interact with Znf179. We also generated the N- (amino acids 1–153) and C-terminal fragments (154–654) of Znf179 and found that the C-terminal but not N-terminal fragment was required for the interaction of Znf179 with Plzf (Figure 3B).

### Effect of Plzf co-expression on subcellular localization of Znf179

**Figure 4:**
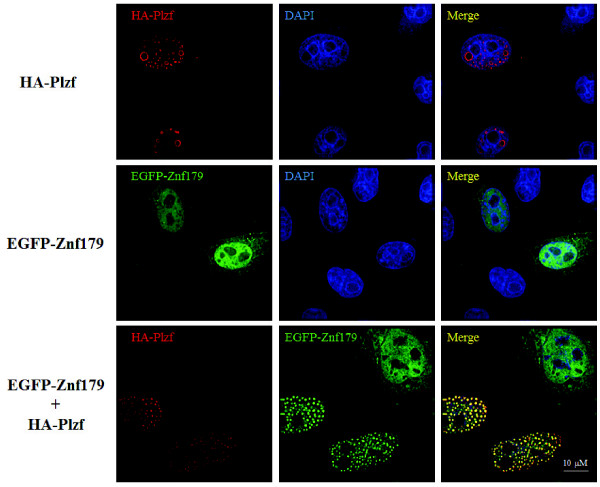
**Overexpression of Plzf changes the sub-cellular localization of Znf179.** HeLa cells were transfected with HA-Plzf, EGFP-Znf179, or HA-Plzf plus EGFP-Znf179 vectors as indicated. After 24 h of transfection, cells were fixed and immunostained with anti-HA antibody (red) followed by DAPI staining (blue).

To further determine the sub-cellular localization of Znf179 and the interaction of Znf179 and Plzf, HeLa cells were transiently transfected with individual constructs or co-transfected with combinations of the HA-tagged Plzf and EGFP-tagged Znf179 constructs and subsequently stained with an anti-HA antibody followed by an immunofluorescence analysis. As shown in Figure 4, Plzf was mostly localized in nuclei and concentrated in nuclear bodies as previous studies reported [28,29], while Znf179 was predominantly localized in nuclei with faint cytoplasmic staining. Interestingly, the co-transfection of Plzf resulted in the recruitment of Znf179 protein from the nucleoplasm to the Plzf-localized nuclear bodies (Figure 4). Taken together, these results indicate that these two proteins indeed interact with each other *in vivo* and the sub-cellular localization of Znf179 is influenced by the expression of Plzf.

### Overexpression of Znf179 does not affect Plzf-mediated transcriptional repression

**Figure 5:**
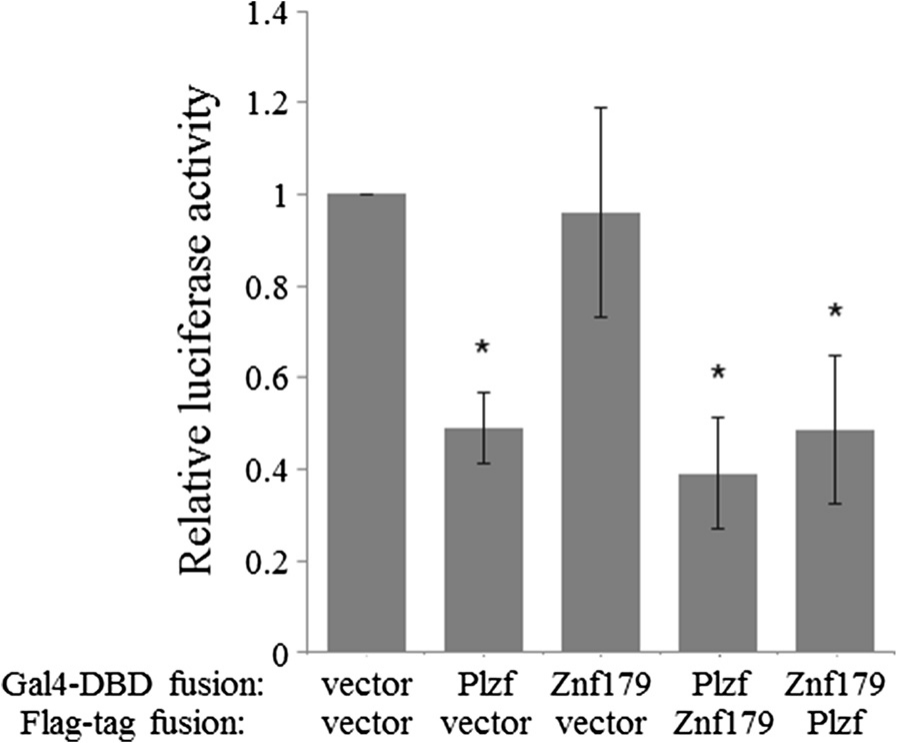
**Effect of Znf179 on the transcriptional repression activity of Plzf.** COS-1 cells were transfected with Gal4-Luc reporter construct (pFR-Luc) and Gal4-DBD-tagged Plzf or Znf179 together with Flag-Plzf, Flag-Znf179, or empty control vector. After 24 h transfection, the cells were collected and subjected to luciferase activity assay. The transfection efficiency was corrected by normalizing the data to the corresponding *Renilla* luciferase activity for each construct. Values of relative luciferase activity represent the mean ± S.E.M. of at least three independent experiments. Statistical analysis was performed using one-way ANOVA with appropriate post hoc tests: *P < 0.05 versus the control (empty vector) group. vector, empty control vector.

Plzf can function as a transcriptional repressor [30]. To examine whether Znf179 affected the transcriptional repression activity of Plzf through protein-protein interaction, we used a Gal4-based transactivation assay. The constructs consisting of Plzf or Znf179, fused with the DNA binding domain (DBD) of the yeast Gal4 transcription factor, were cotransfected with the Gal4 response element-containing luciferase reporter. In agreement with its transcriptional repressor function, our results showed that Gal4-DBD-Plzf inhibited the Gal4 luciferase reporter activity (Figure 5). However, we did not observe a significant difference of Gal4 luciferase reporter activities in cells cotransfected with Gal4-DBD-Plzf and either a control vector or Znf179 expression plasmid. We also found that although Gal4-DBD-Znf179 did not display autonomous transcriptional regulatory activity, the Gal4 luciferase reporter activity was inhibited by coexpression of Plzf (Figure 5), suggesting that Gal4-DBD-Znf179 might recruit Plzf to the Gal4 reporter gene and resulted in inhibition of Gal4 luciferase reporter activity.

**Figure 6:**
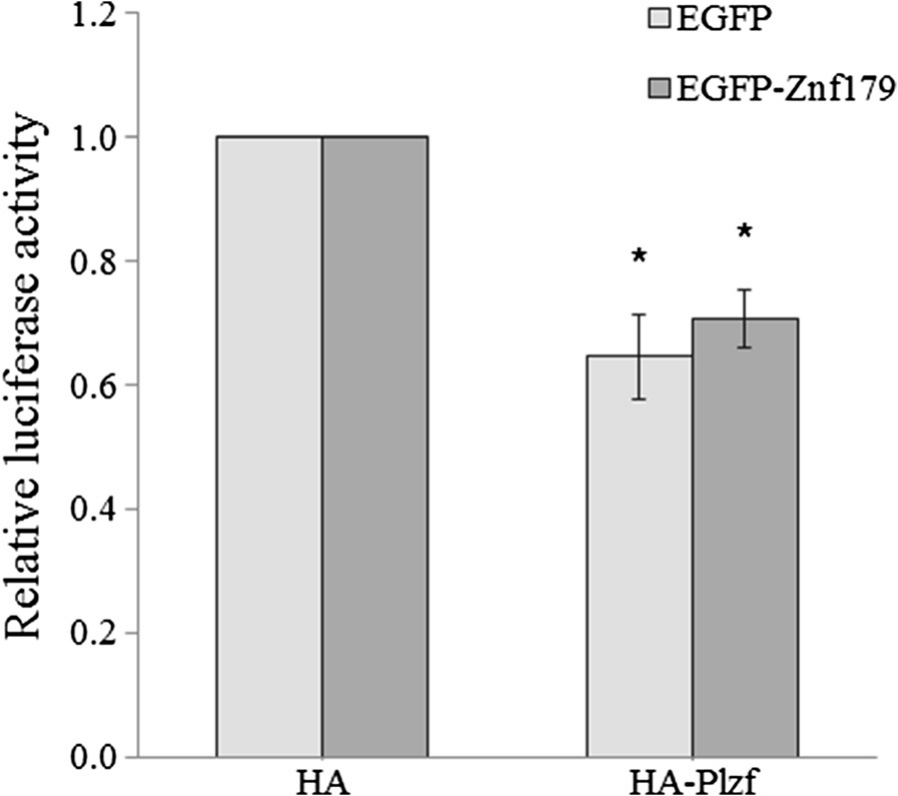
**Effect of Znf179 on Plzf-mediated repression of Aurora kinase C promoter activity.** SW480 cells were transfected with Aurora kinase C promoter construct and HA or HA-Plzf together with EGFP or EGFP-Znf179. After 24 h transfection, the cells were collected and subjected to luciferase activity assay. The transfection efficiency was corrected by normalizing the data to the corresponding *Renilla* luciferase activity for each construct. Values of relative luciferase activity represent the mean ± S.E.M. of at least three independent experiments. Statistical analysis was performed using Student’s *t*-test: *P < 0.05 versus the control (HA vector control) group.

It has been shown that Plzf suppresses aurora kinase C promoter activity in SW480 cells [25]. Therefore, we further examined whether Znf179 affected the transcriptional repression activity of Plzf on aurora kinase C promoter. Our results showed that HA-Plzf inhibited aurora kinase C promoter activity in SW480 cell (Figure 6). However, we did not observe changes in the aurora kinase C promoter activities following cotransfection of Plzf with Znf179 or control vector (Figure 6).

### Znf179 regulates the expression of Plzf at protein level

**Figure 7:**
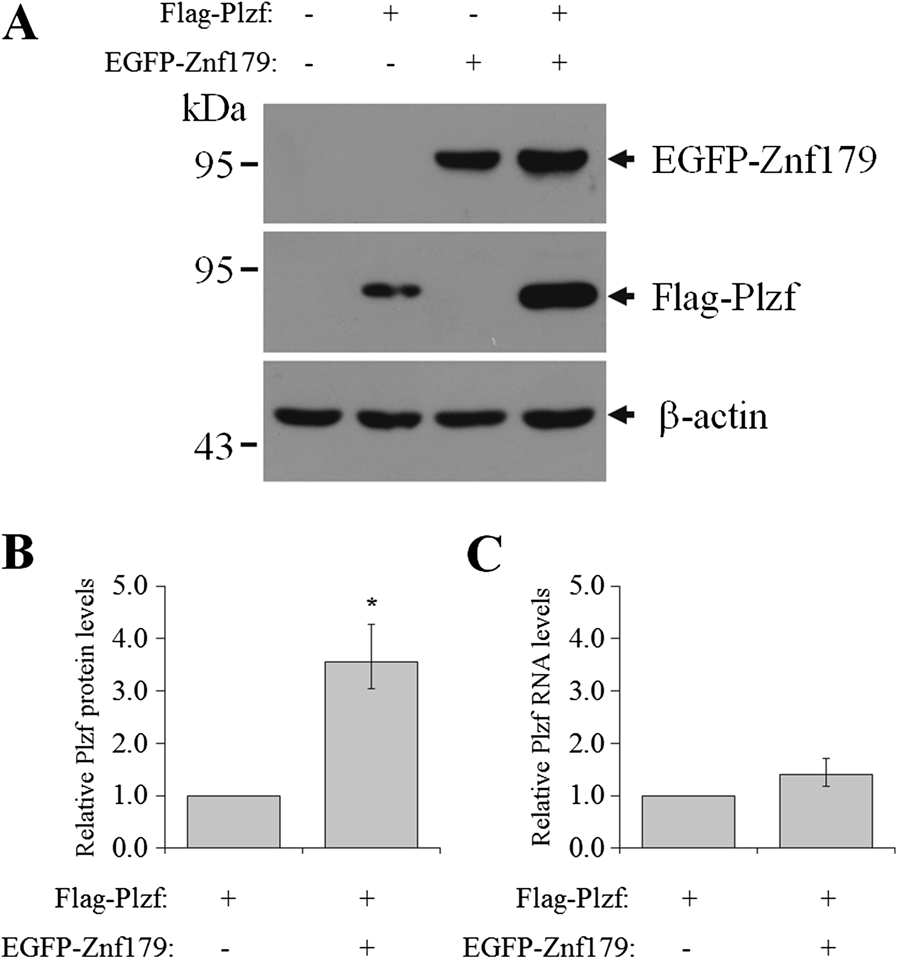
**Znf179 regulates the expression of Plzf at protein level.** COS-1 cells were transfected with EGFP or EGFP-Znf179 together with Flag or Flag-Plzf. **(A)** After 24 h transfection, the expression of Plzf and Znf179 were determined by Western blot analysis. **(B)** Relative amounts of Plzf protein were quantified and normalized to β-actin. **(C)** Relative amounts of Plzf RNA were determined by quantitative RT-PCR and normalized to β-actin. Values represent the mean ± S.E.M. of at least three independent experiments. Statistical analysis was performed using Student’s *t*-test: *P < 0.05 versus the control group.

The stability of Plzf was reported to be regulated by its interacting protein [31]. In that study, Jin and coworkers have demonstrated that KLK4 interacted with Plzf and decreased its protein stability. We therefore examined whether Znf179 interacted with Plzf and contribute to its protein stability. Cotransfection of Znf179 resulted in a significant increase in the protein level of ectopically expressed Plzf (Figure 7A and B). Further analysis by quantitative real-time RT-PCR demonstrated that mRNA level of Plzf was not changed in the presence of Znf179 (Figure 7C). These results suggest that Znf179 interact and regulate Plzf expression at posttranscriptional level.

## Discussion

Znf179 is an evolutionarily highly conserved RING finger protein, suggesting an important function of this gene. In our previous study, we first provide evidence showing functions of Znf179 in neuronal differentiation [3]. The potential function of Znf179 at molecular level is further examined by a yeast two-hybrid screen which has identified Plzf as a Znf179 interacting protein. Our results suggest that the C-terminal but not N-terminal fragment of Znf179 interacts with the first two zinc fingers of Plzf. The result also shows that Plzf possess an autonomous activating activity (Figure 1B), which this autonomous activation of Plzf is consistent to previous report [27]. In that study, Gao *et al*. have found that the C-terminal zinc finger domain is crucial for autonomous activation [27]. Plzf is a transcriptional regulator that can both repress and activate gene expression [9-11,15,16]. The function of Plzf may depend on its interaction partners in cells. In the study of David *et al.*, Plzf represses transcription by recruiting a histone deacetylase through the SMRT-mSin3-HDAC co-repressor complex [14]. In contrast, Plzf is found to activate GATA4 transcription by binding to angiotensin II-activated AT2 receptor [32,33]. Plzf contains an N-terminal BTB/POZ domain and nine *kruppel*-like C2H2 zinc fingers. The N-terminal BTB/POZ domain is required for homo/heterodimerization, nuclear localization, and direct binding of corepressors [12,13]. However, our results showed that the region containing the first two zinc fingers of Plzf is critical for the interaction with Znf179 (Figure 3A). Although zinc finger domains frequently bind DNA, there are many examples in which zinc finger domains participate in protein-protein interactions [34-36]. Previous studies have shown that the region containing the first three N-terminal zinc fingers of Plzf are required and sufficient for Plzf to bind retinoic acid receptor (RAR) [37]. The interaction of Plzf with RAR decreases the ability of RAR to dimerize with retinoid X receptor (RXR) and diminished the transcriptional activity of RAR [37]. The zinc fingers of Plzf are also involved in interaction of Plzf with other proteins, such as GATA2 and proHB-EGF [38,39]. We have also observed that Znf179 interacts with Plzf and results in increase the ectopic expression of Plzf at posttranscriptional level. However, the repressions of Gal4 luciferase reporter and aurora kinase C promoter activity by Plzf are not different in the presence of Znf179 or not. We speculate that, first, the protein level of ectopic Plzf expression in the Plzf-transfected only cells may be enough for the maximal suppression. Second, Znf179 indeed affects the ability of Plzf to regulate aurora kinase C promoter activity. However, the effect of Znf179 on Plzf repression activity is compensated by the increase of Plzf protein. However, it is still possible that Znf179 may affect the ability of Plzf to regulate specific downstream target genes.

Plzf is subject to several different post-translational modifications, including phosphorylation, acetylation and conjugation to ubiquitin and SUMO-1 [24,40-43]. Btbd6a was found to promote the relocation of Plzf from nucleus to cytoplasm and targets Plzf for ubiquitination and degradation [24]. In contrast, the deubiquitinating enzyme USP37 interacts with Plzf which increases Plzf protein stability [44]. In addition, Plzf is found to be phosphorylated by CDK2 on Ser197 and Thr282 and this phosphorylation results in a decrease in protein stability [40]. In our study, we have found that Znf179 interacts with Plzf and increases the ectopic expression of Plzf at posttranscriptional level. It is possible that interaction of Plzf with Znf179 may affect its interaction with other protein and/or alters its post-translational modification, which results in an increase of the Plzf protein.

The expression of the Znf179 gene is restricted to the brain and is regulated during brain development [1,2]. However, the Plzf is widely expressed in neural progenitors and functions to inhibit neurogenesis [24]. The interaction and reciprocal regulation between Znf179 and Plzf during the neurogenesis is an important issue. Znf179 is a RING finger protein with a characteristic C3HC4 motif located in the N-terminus. It is known that many RING finger proteins act as E3 ubiquitin ligases and are associated with the ubiquitin proteasome pathway [45]. In human genome, more than 600 RING finger proteins were annotated as E3s [46]. Whether Znf179 functions as an E3 ubiquitin ligase needs to be further investigated. Our results reveal that Znf179 interacts with Plzf and increased Plzf expression at posttranscriptional level. In other words, if Znf179 functions as an E3 ubiquitin ligase, Plzf may not be its substrate. Plzf is found to be an adaptor of E3 ligase cullin 3 [47]. In the study of Mathew *et al.*, Plzf recruits cullin 3 to the nucleus to alter the ubiquitination pattern of their associated chromatin modifying complex. In our result, we also found that co-expression of Plzf changes the sub-cellular localization of Znf179 from the nucleoplasm to the Plzf nuclear bodies (Figure 4), suggesting that Plzf possibly functions as an adaptor of Znf179. However, the precise nature and role of Znf179-Plzf interaction remain to be elucidated.

## Conclusions

We found that Plzf interacted with Znf179 and recruited Znf179 to the nuclear bodies. Although we did not find that Znf179 could affect the transcriptional repression activity of Plzf in the Gal4-dependent transcription assay system. We can’t rule out the possibility that Znf179 may affect the ability of Plzf to regulate specific downstream target genes. Our findings provide further research directions for studying the molecular functions of the Znf179/Plzf complex.

## Abbreviations

Plzf: Promyelocytic leukemia zinc finger; SMS: Smith-Magenis syndrome; HD: Huntington’s disease; ALS: Amyotrophic lateral sclerosis; APL: Acute promyelocytic leukemia; ATRA: *all-trans*-retinoic acid; HDAC: Histone deacetylase; PCR: Polymerase chain reaction; DBD: DNA binding domain; AD: Activation domain; RAR: retinoic acid receptor; RXR: Retinoid X receptor; IP: Immunoprecipitation; WB: Western blotting.

## Competing interests

The authors declare that they have no competing interests.

## Authors’ contributions

Conceived and designed the experiments: DL, CH and YL. Performed the experiments: YH, HL, PP, JT and CL. Analyzed the data: DL, CH, WC and YL. Contributed reagents/materials/analysis tools: LH and JM. Wrote the paper: DL and YL. All authors read and approved the final manuscript.

## Supplementary Material

Additional file 1: Table S1 Primer list for the plasmid construction. Click here for file
