# Supplementary material for: Analysis of the interaction between Zinc finger protein 179 (Znf179) and promyelocytic leukemia zinc finger (Plzf)
Source: J Biomed Sci. 2013 Dec 20;20(1):98. doi: 10.1186/1423-0127-20-98 (PMC3878200; doi:10.1186/1423-0127-20-98)
Supplement: Additional file 1: Table S1 — Primer list for the plasmid construction. [file 1423-0127-20-98-S1.pdf]

Table S1. Primer list for the plasmid construction

Construct	Forward Primer (5`-3`)	Reverse Primer (5`-3`)	Template
LexA-Znf179 (1-417)	ccgggaattcATGCCGAGGCCCGTCCT	cgcggatccCTAGCTCAGCACGTCCAAGAT	IMAGE 4506141
EGFP-Znf179	gatctcgagCTATGCCGAGGCCCGTCCTGTCAG	ggtggatccCTATTCCTCCTGGAGTAGGGGCTCTCG	IMAGE 4506141
EGFP-Znf179 (1-153)	gatctcgagCTATGCCGAGGCCCGTCCTGTCAG	ccggaattcctaGGTCTCCTGCAGTGC	IMAGE 4506141
EGFP-Znf179 (154-654)	ccggaattcatgTGTGCTGTGAGGGCAGAA	ggtggatccCTATTCCTCCTGGAGTAGGGGCTCTCG	IMAGE 4506141
Flag-Plzf	cgcggatccgcATGGATCTGACAAAAATG	ccgctcgagTCACACATAGCACAGG	IMAGE 4944546
Flag-Plzf (1-398)	cgcggatccgcATGGATCTGACAAAAATG	ccgctcgagCGCTACTCTGACTTCATGCC	IMAGE 4944546
Flag-Plzf (180-673)	cgcggatccATGGTGGACCAGAGCCCT	ccgctcgagTCACACATAGCACAGG	IMAGE 4944546
Flag-Plzf (398-673)	cgcggatccATGAGCCGGACCATCGGA	ccgctcgagTCACACATAGCACAGG	IMAGE 4944546
Flag-Plzf (455-673)	cgcggatccATGGCGGGTGCCAAAGCC	ccgctcgagTCACACATAGCACAGG	IMAGE 4944546
Flag-Plzf (515-673)	cgcggatccATGCGCAGCTACATCTGC	ccgctcgagTCACACATAGCACAGG	IMAGE 4944546
